# Mosquito control by abatement programmes in the United States: perspectives and lessons for countries in sub-Saharan Africa

**DOI:** 10.1186/s12936-023-04829-3

**Published:** 2024-01-04

**Authors:** Eric Ochomo, Samuel S. C. Rund, Rosheen S. Mthawanji, Christophe Antonio-Nkondjio, Maxwell Machani, Siriman Samake, Rosine Z. Wolie, Sandrine Nsango, Laurel Anne Lown, Damaris Matoke-Muhia, Luna Kamau, Edward Lukyamuzi, Jane Njeri, Joseph Chabi, Otubea Owusu Akrofi, Charles Ntege, Victor Mero, Charles Mwalimu, Samson Kiware, Etienne Bilgo, Mohamed Moumine Traoré, Yaw Afrane, Emmanuel Hakizimana, Mbanga Muleba, Emma Orefuwa, Prosper Chaki, Elijah Omondi Juma

**Affiliations:** 1 Entomology Department, Centre for Global Health Research, Kenya Medical Research Institute, Kisumu, Kenya; 2 Vector Control Products Unit, Researchworld Limited, Kisumu, Kenya; 3 University of Notre Dame, Notre Dame, IN USA; 4 Vector Biology Group, Malawi Liverpool Wellcome Trust Clinical Research Programme, Blantyre, Malawi; 5 Organisation de Coordination Pour la lutte contre les Endémies en Afrique centrale (OCEAC), Yaounde, Cameroon; 6 University of Clinical Research Center, Bamako, Mali; 7 Vector Control Product Evaluation Centre - Institut Pierre Richet (VCPEC-IPR), Institut National de Santé Publique (INSP), Bouaké, Côte d’Ivoire; 8 Unité de Formation et de Recherche des Sciences de la Nature, Université Nangui Abrogoua, Abdijan, Côte d’Ivoire; 9 Faculty of Medicine and Pharmaceutical Sciences, University of Douala, Douala, Cameroon; 10 Centre Pasteur in Cameroon, Yaounde, Cameroon; 11 Pan-African Mosquito Control Association (PAMCA), KEMRI Headquarters, Nairobi, Kenya; 12 Centre for Biotechnology Research and Development, Kenya Medical Research Institute, Nairobi, Kenya; 13 Abt Associates, Rockville, DC USA; 14 National Malaria Elimination Programme (NMEP), Accra, Ghana; 15 National Malaria Control Division Ministry of Health, Kampala, Uganda; 16 Ifakara Health Institute (IHI), Dar es Salaam, Tanzania; 17 National Malaria Control Programme, Dar es Salaam, Tanzania; 18 Institut de Recherche en Sciences de la Sante (IRSS) Direction regionale de l’Ouest, Bobo Dioulasso, Burkina Faso; 19 Malaria Research and Training Centre, Faculty of Medicine, Pharmacy and Odonto-Stomatology, University of Sciences, Techniques and Technology of Bamako, BP 1805, Bamako, Mali; 20 Department of Medical Microbiology, University of Ghana Medical School, College of Health Sciences, University of Ghana, Accra, Ghana; 21 Malaria and Other Parasitic Diseases Division, Rwanda Biomedical Centre (RBC), Ministry of Health, Kigali, Rwanda; 22 Pan-African Mosquito Control Organization (PAMCO), Rwanda Chapter, Kigali, Rwanda; 23 Tropical Diseases Research Centre, Ndola, Zambia

**Keywords:** Mosquito control, Mosquito abatement districts, Larval source management, Surveillance, Integrated vector management

## Abstract

Africa and the United States are both large, heterogeneous geographies with a diverse range of ecologies, climates and mosquito species diversity which contribute to disease transmission and nuisance biting. In the United States, mosquito control is nationally, and regionally coordinated and in so much as the Centers for Disease Control (CDC) provides guidance, the Environmental Protection Agency (EPA) provides pesticide registration, and the states provide legal authority and oversight, the implementation is usually decentralized to the state, county, or city level. Mosquito control operations are organized, in most instances, into fully independent mosquito abatement districts, public works departments, local health departments. In some cases, municipalities engage independent private contractors to undertake mosquito control within their jurisdictions. In sub–Saharan Africa (SSA), where most vector-borne disease endemic countries lie, mosquito control is organized centrally at the national level. In this model, the disease control programmes (national malaria control programmes or national malaria elimination programmes (NMCP/NMEP)) are embedded within the central governments’ ministries of health (MoHs) and drive vector control policy development and implementation. Because of the high disease burden and limited resources, the primary endpoint of mosquito control in these settings is reduction of mosquito borne diseases, primarily, malaria. In the United States, however, the endpoint is mosquito control, therefore, significant (or even greater) emphasis is laid on nuisance mosquitoes as much as disease vectors. The authors detail experiences and learnings gathered by the delegation of African vector control professionals that participated in a formal exchange programme initiated by the Pan-African Mosquito Control Association (PAMCA), the University of Notre Dame, and members of the American Mosquito Control Association (AMCA), in the United States between the year 2021 and 2022. The authors highlight the key components of mosquito control operations in the United States and compare them to mosquito control programmes in SSA countries endemic for vector-borne diseases, deriving important lessons that could be useful for vector control in SSA.

## Background

The history of mosquito control is a history of human beings’ dalliance with “the world’s most dangerous animal” [1]. Mosquito control in sub-Saharan Africa and the continental United States have followed disparate paths, motivated by different goals over the course of time. Historically, mosquito control programmes in sub-Saharan African (SSA) countries have been primarily motivated by the need to control mosquito-borne diseases, mostly malaria. As such, the programmes are characterized by interventions that, by and large, target the adult vectors within the home environment (where majority of the disease transmission has been shown to occur, to disrupt community-wide transmission) [2, 3]. For over three decades now since the early 1980s, mass distribution of long-lasting insecticidal bed nets (LLINs) and indoor residual spraying (IRS), have remained the mainstay of vector control programmes in vector-borne disease-endemic SSA countries (IRS) [4–6].

Even with significant reduction in malaria burden in SSA over the last two decades since the year 2000, the SSA still accounts for up to 95% of global malaria morbidity and mortality cases [7]. This reduction has been primarily attributed to mass distribution of bed nets and IRS campaigns targeting endemic populations [5]. These tools have been demonstrated to be operationally and logistically feasible to distribute in campaigns and through routine channels supported by infrastructure established over the years [8–10]. As a results, the pace of adoption of other mosquito control interventions as part of an integrated vector management (IVM), such as larval source management (LSM) has been very slow. Whereas many of the vector-borne disease endemic SSA countries do have LSM encoded in their vector control operational guidelines as part of their IVM policies, LSM has received limited traction due to a myriad of factors including, WHO’s recommendation as a supplementary intervention which impedes the ability of the national programmes to mobilize funding resources; with major donors, such as Global Fund due to perceived limited viability under operational conditions [11]. With the waning efficacy of the conventional tools (LLINs and IRS) due to emergence and spread of insecticide resistance and the prohibitive cost of expanding IRS, especially with new actives [12, 13] implementing LSM as a complementary intervention to LLINs and IRS, within an integrated vector management framework, could provide added benefit to disease control programmes by targeting outdoor and day biting vectors [14]. Additionally, LSM targets all aquatic stages (thus reducing both disease and nuisance vectors) and more particularly targeting urban aquatic habitats of the invasive *Anopheles stephensi* currently colonizing many countries in SSA [15, 16].

In the United States, mosquito control programmes have evolved remarkably from the early twentieth century to date, spanning three major phases: (1) mechanical control era, characterized largely by ditching and drainages, with limited application of oil and Paris green for larviciding operations (1900–1942); (2) chemical control era, catalysed by the discovery of insecticidal properties of DDT, and house screening (1942–1972); (3) integrated control era, ushered in following widespread development of resistance to DDT by most of the targeted mosquito vectors, and the Environmental Protection Agency’s (EPA) ban on DDT due to its adverse toxicological effects on humans and animals, and the environment generally. This era coincided with the development of more environmentally safe synthetic pyrethroids to replace DDT and maintain efficacy in control. There was also a shift to more environmentally friendly biopesticides [e.g. *Bacillus thuringiensis* var. israelensis (*Bti*) and *Lysinibacillus sphaericus* (*Bacillus sphaericus)*], and intense research on and application of other integrated approaches (1972–present) [1, 17]. This evolution came alongside the creation of decentralized mosquito abatement programmes organized at state, or city/county levels, governed and funded through different models.

Beginning the year 2021, the Pan-African Mosquito Control Association (PAMCA) initiated an exchange programme with American Mosquito Control Association (AMCA) members’ mosquito abatement districts, in efforts coordinated by the University of Notre Dame. This exchange programme is part of initiatives aimed at operationalizing the collaborative partnership between PAMCA and AMCA on knowledge and experience sharing on mosquito control best practices and capacity enhancement for mosquito surveillance and control between the two continents. In this article, the authors document observations, experiences, and lessons gathered by the African vector control professionals that participated in exchange visits to thirteen mosquito abatement districts across six states.

The authors contend that lessons documented from the thirteen mosquito abatement programmes do not necessarily represent the entire length and breadth of mosquito control programmes in the United States, thus the statements made in this article should not be interpreted in the broad context of the entire United States. However, the authors posit that the exchange programme has been valuable in promoting sharing of knowledge and ideas on advances in mosquito control programmes between the US and SSA, and the two continents have a lot to learn and borrow from each other on best practices on mosquito control that speak to their specific country and continental contexts. The authors make efforts to analyse and compare mosquito control programmes in the US, with their counterparts in countries in SSA. The authors document their governance systems and funding structures, areas of overlap in best practices, and analyse how mosquito control professionals from across the two continents can leverage their diverse expertise and optimize resources to strengthen mosquito surveillance and control operations borrowing from each other.

### Mosquito control in the United States: the present

The continental US experiences less intense mosquito-borne disease transmission attributed to a myriad of factors including unfavourable ecology for more efficient vectors,such as *Anopheles gambiae *sensu lato (*s.l.*), and *Anopheles funestus* group, unfavourable climate with cold winter seasons that disrupts year-round mosquito activity, forcing the mosquito species to overwinter, almost complete elimination of mosquito activity indoors, thus reducing significantly any chance of indoor transmission; well-funded and well organized health systems, and the lower efficiency of the existing mosquito vectors of diseases [18–20]. Nonetheless, the mosquito abatement programmes still deal with a number of disease vectors that transmit disease pathogens in hypoendemic settings, with occasional epidemics being reported. Mosquito-borne pathogens include the relatively high burden West Nile virus (WNV) [21, 22] and St. Louis encephalitis virus (SLEV) [23, 24], both transmitted by *Culex* species (*Culex restuans*, *Culex pipiens* complex, *Culex tarsalis*) in the whole of continental United States. Others of concern include eastern equine encephalitis virus (EEEV) transmitted through infectious bites of *Culiseta melanura* and other bridge vectors, such as *Aedes* or *Coquillettidia* species [25], La Crosse Virus (LACV) transmitted by the eastern tree-hole mosquito, *Aedes triseriatus *[26, 27], and other aedine (e.g. *Aedes aegypti*—with a predicted growing range) borne viruses, such as dengue and chikungunya that are common in the sub-tropical south, and western states of the US [19, 28, 29]. Indeed, many of these disease vectors are several magnitudes lower in their vectorial capacity when compared to their SSA counterparts in the *Anopheles* genus [30]. As of writing this paper, locally-transmitted malaria (*Plasmodium vivax*) has been reported in the continental US in the states of Florida and Texas [31]. This situation raises an alarm on the real danger that countries that have long eliminated malaria face, and the need to remain vigilant with sustained strong surveillance systems to prevent re-introduction.

The surveillance systems, the tools and technologies applied to mosquito control have evolved in scale and sophistication to reflect cumulative advances on mosquito control policies and practices. Mosquito control operations have evolved from blanket application of insecticides to an exact science where mosquito control intervention decisions are made based on carefully calibrated metrics informed by many years of accumulated research knowledge, and up-to-date mosquito surveillance data and information in each abatement district visited [28]. Routine surveillance as a core part of the interventions is conducted by all the programmes visited and involves longitudinal and overnight mosquito collection traps set at established sentinel sites. Mosquito collections and species identifications are conducted at the district laboratories by qualified staff. All the districts visited have moved from paper-based data capture, to capturing surveillance data on integrated geographic information system (GIS) digital databases that facilitate real-time and seamless visualization of granulated surveillance data by trap types, locality, dates, collector, on dashboards for easy analysis and informed and expedited operational decision-making. Some of the cloud-based GIS platforms used include FieldSeeker^®^ and MapVision^®^ and are additionally useful for mapping the dynamics in mosquito aquatic habitats, thus informing surveillance and intervention decisions [32].

Depending on the disease vectors in the districts, WNV testing is done for pooled samples using polymerase chain reaction (PCR) for WNV monitoring and reporting according to state and Centres for Disease Control and Prevention (CDC) regulations and requirements [33]. Some of the districts visited keep insectaries where colonies of local mosquito species are reared for insecticide resistance testing as well as arbovirus monitoring studies. Chemical treatment decisions for larval and adult populations are informed by surveillance data and applications are made based on standardized threshold informed by mosquito trap counts as well as local policies and ordinances guiding environmental application of pesticides [34]. Chemical application equipment is carefully calibrated to ensure that only the exact amount of chemicals is dispensed from the spray equipment, in the right spray location, with the right droplet size, to ensure judicious use of the right chemicals to safeguard against potential environmental harm. Surveillance operations also include monitoring of invasive species, such as *Aedes albopictus* and *Ae. Aegypti,* to keep tabs on their introductions in new states and regions and rate of invasion countrywide. As part of the integrated mosquito management approach, some districts visited have predator fish-rearing programmes for biological control of mosquitoes in confined container habitats. The common species of fish used in the programmes include *Gambusia affinis* (western mosquito fish) and *Gambusia holbrooki* (Eastern mosquito fish). These fish species are widely distributed to homeowners to use in container environments within the residential areas to control mosquito larval growth in such environments [35]. This is part of the district partnership with the district residents in mosquito control. Ecological management through larval source reduction are also practiced widely. The districts may partner with other public works agencies to create lotic drainages or ditches, or entirely remove potential larval habitats. These initiatives aimed at manipulating and modifying the environments, in some cases, eliminate the need for chemical applications and, therefore, make the mosquito control practices environmentally sustainable.

### Funding, organizational models, and governance systems, of mosquito abatement programmes in the US

The mosquito abatement programmes are community-wide public health programmes that seek to protect the health and enhance the quality of life of residents and visitors. The programmes vary in geographical size, budget, administration, and scope (Table 1). It did not escape the authors of this article that the annual per person costs of mosquito control in many districts implementing IVM was under $10, and three were $5 and under. Putting this into context, especially with the oversupply of labour in SSA, comparatively lower labour costs compared to the US, it certainly offers the possibility that implementing community-wide LSM operations as part of broader IVM programmes in SSA might not be nearly as costly as previously assumed [14]. The authors note that they only visited a very small subset of the approximately 1000 governmental units [36] with a role in mosquito control, and that these districts have been found to vary quite dramatically in capacity [37].

**Table 1:** A description of the mosquito abatement programmes: their ecologies, vectors, budgets and populations covered

Abatement District	Square miles	Population	Ecology	Vectors	VBD of note	Total Annual Budget,USD (personnel budget)	Budget/resident (USD)
Sacramento-Yolo	2000	2,000,000	Suburban and Agricultural areas	*Culex tarsalis*	West Nile Virus	18 Million ($12 Million)	9
				*Culex pipiens*			
				*Aedes aegypti*			
Salt Lake City Mosquito Abatement District	110	1,000,000	Rural	*Culex tarsalis*	(−)	7 Million ($2.2 Million)	7
				*Aedes dorsalis*			
			Urban	*Culex pipiens*			
				*Aedes sierrensis*			
Toledo Area Sanitary District	600	425,000	Urban and conservation refuges	*Culex* spp	(−)	3 Million (0.4 Million)	7
Bay County Mosquito Control	442	104,000	Rural, Urban and Suburban	*Anopheles* spp	(−)	1.6 Million (0.3million)	15
				*Coquillettidia perturbans*			
				*Aedes vexan*			
Manatee County Mosquito Control District	750	420,000	Urban	*Aedes taeniorhynchus*	(−)	10 Million (1.5 million)	24
				*Aedes aegypti*			
			Rural	*Psorophora spp*			
Jacksonville Mosquito Control	840	1,100,000	Urban, suburban and Rural	*Aedes* spp	(−)	2.3 Million (0.25 million	2
				*Culex* spp			
South cook county mosquito abatement district	340	1,700,000	Suburban	*Culex* spp	(−)	5.99 Million (0.42 million)	4
			Wooden areas	*Aedes sollicitans*			
				*Aedes albopictus*			
Collier Mosquito Control District	401	2,500,000	Urban and Mangrove swamps	*Aedes taeniorhynchus*	(−)	24 Million (3.5 million	10
				*Psorophora columbiae*			
				*Culex nigripalpus*	West Nile Virus		
				*Culex quinquefasciatus*			
				*Aedes aegypti*	Dengue, Zika and Chikungunya		
				*Aedes albopictus*			
Florida Keys Mosquito Control District	140	75,000	Private and public land	*Aedes aegypti*	Dengue	15 Million (2.5 million)	200
				*Aedes taeniorhynchus*			
North Shore Mosquito Abatement District	69	330,000	Urban and Suburban	*Culex pipiens*	(−)	1.62 Million (0.25 Million)	5
				*Culex restuans*			
				*Aedes vexans*			
East Flaglier Mosquito Control District	117	100,000	Salt Marsh		(−)	3.5 Million (0.5 million)	35
Anastasia Mosquito Control District	603	400,000	Flood water and salt marsh	*Culex quinquefasciatus*	(−)	6.5 Million (1 million)	16
				*Culex nigripalpus*	(−)		

(-) means no response, meaning for these districts, their primary focus was nuisance mosquito control

Within the current ambit of mosquito control programmes in the US, three main organizational models are recognized namely, (1) mosquito abatement districts which are independent taxing units, and thus have autonomous funding and control. The mosquito abatement district might cover the whole county geographic area, or some counties may have more than one mosquito abatement district; (2) mosquito control programmes embedded within the city, or county public health or public works departments underwritten through city or county budgets; (3) independent mosquito control contractors formally engaged by states, or local municipalities to conduct mosquito control activities. Stand-alone mosquito abatement districts were the majority of programmes visited] [38]. Under this model, each mosquito abatement district is managed by a director or district manager, the principal administrative and technical head of the programme, overseen by a board which is elected directly or indirectly by residents of the district’s jurisdiction. The governance framework also consists of technical staff involved in day-to-day mosquito control operations including, mosquito surveillance (field and laboratory), equipment maintenance, procurement, human resource and administration. The abatement districts work in partnership with other organizations, including professional organizations or state agencies, mosquito control associations, state technical advisory committees, other abatement programmes, fish and wildlife services and universities. Each of these organizations play a key role including setting operational guidelines and standards for treatment decisions, receiving, reviewing and updating evidence on mosquito control best practices, and advancing the profession, among others. Operations of the mosquito abatement districts are covered by specifically ring-fenced budgets, or funds collected from taxes paid by each resident of the district, with mosquito control being a specific budget line item on the tax bill. Often, these taxes are proportionate to the value of the property (land/house) of the resident (*e.g.,* ‘property tax’ or ‘millage’). The mosquito control budget typically covers expenses such as: personnel, mosquito control supplies (chemical insecticides), capital equipment (treatment equipment and machinery), daily surveillance operations, wages/fringes for the personnel, and other services [38].

### Mosquito control programmes in sub–Saharan Africa: national malaria control/elimination programmes (NMCP/NMEP)

The presence of globally acclaimed ‘world’s most efficient vectors’, *An. gambiae* complex and *An. funestus* group in SSA facilitate an efficient malaria transmission ecosystem [39]. This, coupled with a conducive hot and humid climate that facilitates year-round mosquito activity, under-resourced health infrastructure, and funding challenges, result in mosquito-borne disease burden as a major public health challenge [40]. Malaria control is the primary driver of vector control activities and is managed by NMCP/NMEP that are domiciled within the Ministries of Health (MoH) headquarters at the national or federal level. The agencies are responsible for formulating vector control policies. However, the process is rarely devolved to the sub-national level where vector control activities are implemented at the last mile. The agencies are overseen by a programme head (Programme Director) working with a minimal number of technical and administrative staff. The programmes’ core operations and budget are in malaria case management with the distinct operations having limited linkage with other vector-borne diseases, such as the arboviral vector control programmes even when they occur within the same locality. The case management (diagnosis and treatment), vector, and epidemiological surveillance operations typically exceed its technical staff capacity [41]. In contrast, the US offers an alternative model from SSA model for vector control to be considered. One that features standalone decentralized programmes that are independently run and funded with specific vector control objectives and budgets, with technical capacity for sub-national tailoring and consideration applied through an integrated vector management (IVM) approach based on their ecologies, data, and budgets.

To facilitate a concerted decision-making process, many of these SSA vector control programmes have technical working groups (TWGs) and committees of experts that are composed of technical professionals drawn from local research, academic, private, civil society, and partner funding organizations, based in-country, that assist in policy formulation and technical guidance. The TWGs, or committees of experts, as they are referred to in some cases, are convened by the programmes on a quarterly basis, or on an ad-hoc basis [42]. These programmes rely on WHO technical guidelines and recommendations for vector control and are, to a great extent, dependent on donor funding, to supplement the limited national budget (mostly supporting salaries and administration). For this reason, the programmes have remained largely prescriptive and one-size-fits-all. The recommendations and guidelines are largely tailored to the two main vector control interventions, LLINs and IRS. Due to the limited human and resource capacity in most of the malaria-endemic countries in Africa, routine vector surveillance activities are conducted in a few sentinel sites, thus creating gaps in countrywide surveillance operations. Some of these programmes include additional interventions, such as LSM in their vector control guidelines as part of the overall IVM framework, but LSM has received little attention because of its perceived operationally intensive nature, requirement for highly qualified technical staff, limited funding from multilateral donor agencies that fund vector control operations in SSA such as the Global Fund and PMI [14, 16], and reluctance by the policy makers to advocate for it due to lack of clear and supportive policy guidelines from the WHO [43].

However, it is important to note that prior to the era of mass LLIN distribution and IRS campaigns (the early 1980s), a number of African countries implemented LSM at operational scale to counter *An. gambiae s.l.* populations and consequently reduce or eliminate malaria transmission. In Egypt, LSM was applied to eliminate *An. gambiae* in the Upper Nile Region by 1945 using Paris Green [44]. Similarly, in Zambia, in the copper mining belt, an LSM programme primarily focused environmental management (habitat modification and larval source reduction) was implemented resulting in a documented 70–95% reduction in malaria incidence between 1929 and 1950 [45] A similar environmental management programme was implemented in along the malaria-endemic coastal regions of Nigeria between 1942 and 1943 reporting up to 77% reduction in malaria incidence during that period [46]. With the growing need to expand the vector control toolbox, and the demonstrated efficacy of LSM in a number of LSM in recent studies in SSA, few countries, such as Ghana, Kenya, Tanzania, Rwanda, and Cameroon, have started implementing LSM as part of the national vector control operational guidelines within the context of the IVM framework [47–49]. These are currently implemented at smaller scale, on pilot basis, with locally generated exchequer funding, to generate additional evidence on operational feasibility of implementing LSM in the countries in specific viable contexts but are often under-resourced, and poorly implemented and monitored [47–49].

### PAMCA delegation tours of US mosquito abatement programmes

Between February 2021 and July 2022, twenty-four individuals representing various SSA countries’ national malaria programmes and staff, public health entomologists, researchers, academics, and students with membership to PAMCA participated in visits to several US mosquito abatement programmes to learn about mosquito control practices in the US, document lessons that could be contextualized, and applied to SSA countries’ mosquito control contexts, and to explore opportunities for collaboration in SSA-US mosquito district through twinning programmes. The list of mosquito abatement districts toured are indicated in Table 1. In total, the teams separately visited twelve districts (and one corporate provider of integrated pest management (IPM) service to contracting municipalities) in six states of the US. The visits exposed the delegations to a diversity of local economic and political landscapes, varied organizational and management structures, programme operational scales, sizes, and focus, as well as local mosquito control solutions and implementation approaches. Ten out of the twelve districts visited (83%) are fully autonomous and two are entities anchored within the municipal government. The team observed diverse ecological contexts and thus mosquito vector diversities, ranging from predominantly urban, to rural/farmland, and marshlands, in both temperate and sub-tropical climates across the six states visited.

For further exposure to the diversity of mosquito control solutions in the US context, delegations also attended either the 93rd Florida Mosquito Control Association (FMCA) meeting at the Florida Keys, Florida (2021), or the 88th AMCA Annual Meeting in Jacksonville, Florida (2022). The mosquito abatement programmes in the US have a strong local, regional, and nation-wide partnership and collaborations through professional membership to local or multi-state mosquito control associations, and at the national level as the AMCA. This organizational architecture has provided a platform since 1938 for its membership to congregate annually to network, share research knowledge and advances on best practices on mosquito surveillance and control practices and to keep the mosquito community informed on new tools, technologies, regulatory practices, among others. Besides individual membership, mosquito abatement programmes also subscribe to AMCA as corporate members and, therefore, work in concert to advance the field of mosquito control in the US [50].

### Mosquito abatement district survey

**Fig. 1:**
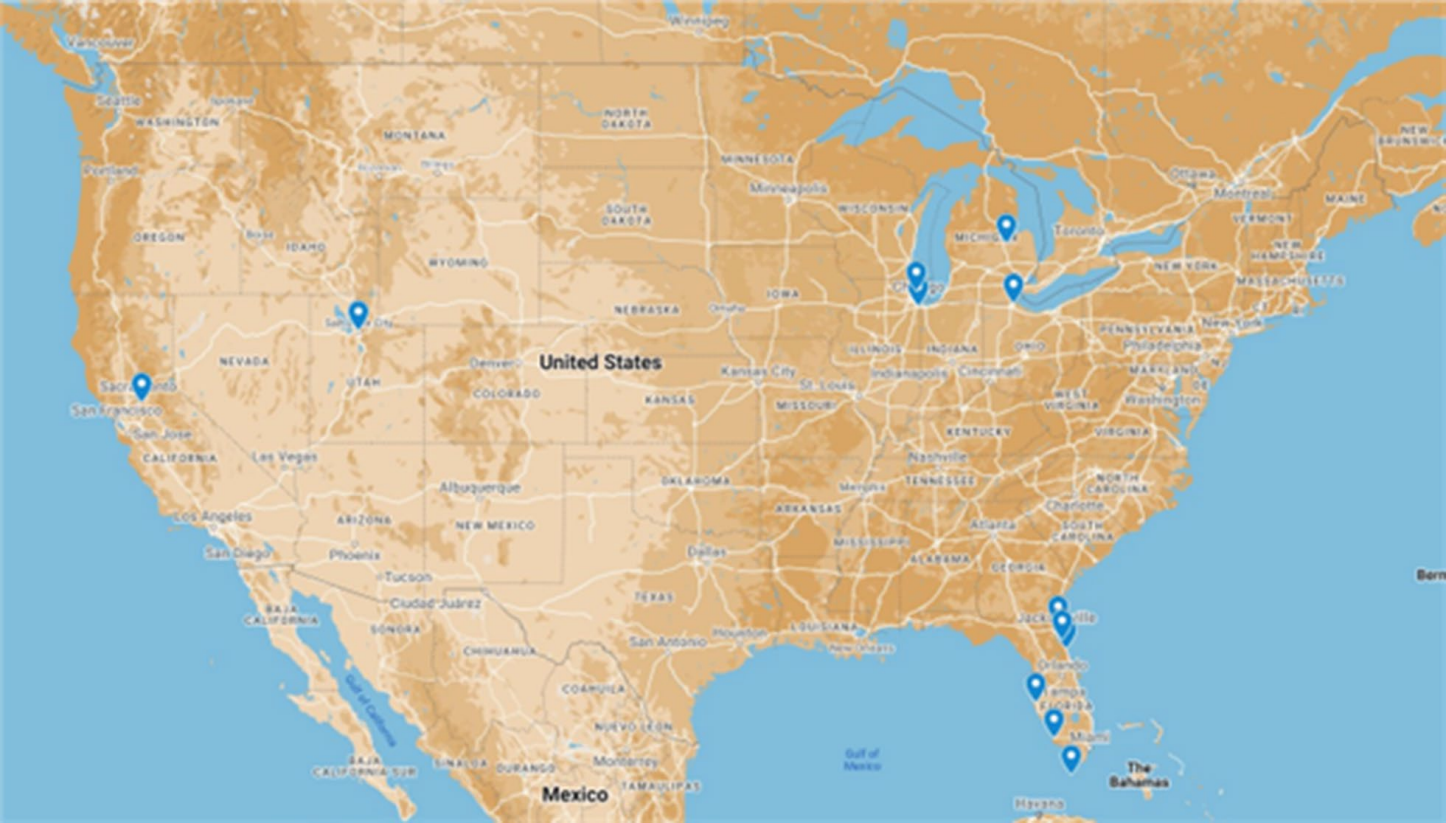
Map of the United States showing the locations of the abatement programmes visited by the delegation from PAMCA

Following the tour of the mosquito abatement programmes a survey of the districts that participated in the learning tours across the US was conducted (Fig. 1). It is noted that the delegation only visited a very small subset of the approximately 1000 governmental units with some sort of role in mosquito control, and that these districts have been found to vary quite dramatically in capacity [28].

The goal of the survey was to get more in-depth context into mosquito surveillance and control practices in the US from the participating programmes. Key parameters of interest captured in the survey included: district geographic size, the population size of communities served within the district jurisdiction, the scale and type of tools and equipment used in mosquito surveillance, the ecological dynamics of the district, annual budgets, and length of control season as illustrated in Tables 1, 2.

**Table 2:** Equipment and personnel at each abatement programme

Abatement district name	Square miles	Drones pesticide application	Helicopters and/or airplanes	Trucks	Full time employees	seasonal employees	Length of control season (Months)
Sacramento-Yolo MVCD	2000	Yes	Yes	98	70	25	9
Salt Lake City Mosquito Abatement District	110	Yes	Yes	25	10	33	7
Toledo Area Sanitary District	600	No	No	37	19	13	8
Bay County Mosquito Control	442	No	No	33	7	32	8
Manatee County Mosquito Control District	750	No	Yes	25	27	3	12
Jacksonville Mosquito Control	840	No	Yes	27	24	4	8
SCCMAD South Cook County Mosquito Abatement District	340	No	No	55	24	22	7
Collier Mosquito Control District	401	Yes	Yes	33	49	2	12
Florida Keys Mosquito Control District	140	No	Yes	30	70	0	12
North Shore Mosquito Abatement District	69	No	No	18	7	16	8
East Flagler Mosquito Control District	117	Yes	Yes	22	12	5	12
Anastasia Mosquito Control District	603	No	Yes	20	31	11	12

#### Tour 1

In February 2021, three individuals visited the North Shore Mosquito Abatement District (NSMAD) in the greater Chicago, Illinois area; Florida Keys Mosquito Control District (FCMCD), Florida; Manatee County Mosquito Control (MCMCD); Collier Mosquito Control District (CMCD); Anastasia Mosquito Control District of St. Johns County (AMCD), and attended the Florida Mosquito Control Association (FMCA) Annual Meeting; and finally, the Salt Lake City Mosquito Abatement District (SLCMAD) in Salt Lake City, Utah.

#### Tour 2

In November 2021, nine members of the PAMCA delegation visited AMCD; East Flagler Mosquito Control District (EFMCD); and the City of Gainesville Mosquito Control District (CGMCD) all in Florida. Afterwards, the delegation attended the American Mosquito Control Association (AMCA) Annual Meeting. During this leg of the tour the delegation also visited a demonstration site of unmanned aerial vehicle (UAV) (drones) technologies by Leading Edge Aerial Technologies, leading experts in UAV technologies for aerial applications, who staged an extensive demonstration of their numerous drone-based mosquito adulticide and larvicide solutions.

#### Tour 3

**Fig. 2:**
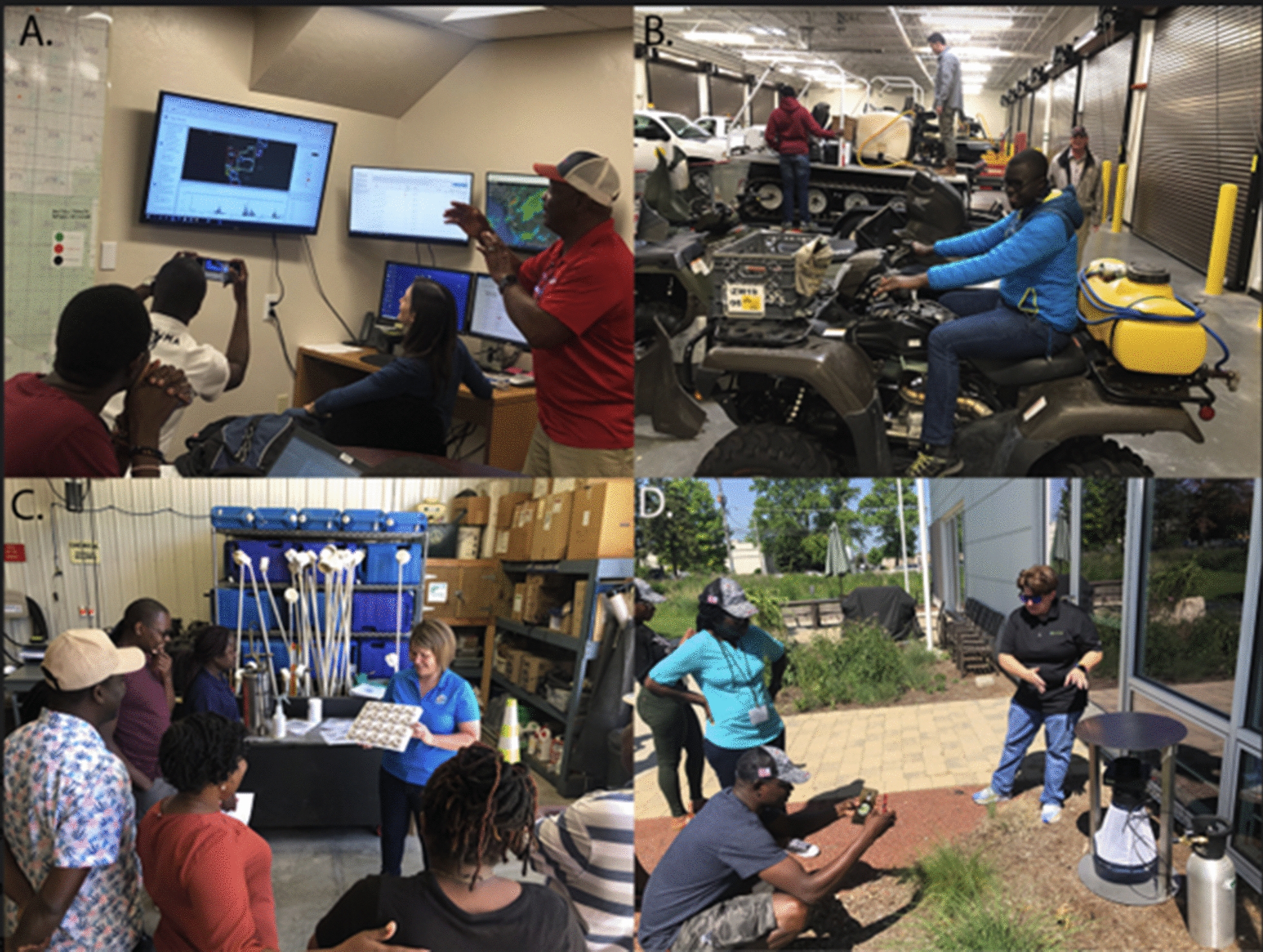
**A**. Operations room of Collier County Mosquito Control where surveillance reports are received, and control operations are planned **B**. Diversity of vehicles required for LSM activities at Salt Lake Country Mosquito Control District. **C**. Rebecca Brandt, of Bay Shore County Mosquito Control demonstrates of larval control and surveillance **D**. Clarke Mosquito Control demonstrates smart, web-connected mosquito traps used for operational decision-making

In June 2022, thirteen members of the PAMCA delegation visited the South Cook County Mosquito Abatement District (SCCMAD); Clarke Mosquito Control (Clarke Mosquito Control serves as both a supplier of mosquito control products, but also as an operational mosquito control service provider contracted by municipalities to provide all mosquito control services in a given jurisdiction); and North Shore Mosquito Abatement District (NSMAD) in Chicago, Illinois. Eight members of the delegation further visited Toledo Area Sanitary District (TASD), Toledo, Ohio; and Bay County Mosquito Control (BCMC) in Bay County, Michigan (Fig. 2).

#### Tour 4

In July 2022, PAMCA sent two staff members of the NMCPs of Tanzania and Ghana for an extended three weeks visit of the Sacramento-Yolo Mosquito and Vector Control District (SYMVCD), California. The goal was for the two staff members, who are also the head of entomology unit in their programmes, to have a more intensive interaction with the SYMVCD to closely observe the day-to-day operations of the district. The two national programmes of the two countries, Ghana and Tanzania, were specifically selected to participate in the visit because these two countries have initiated limited capacity in LSM and have expressed interest in scaling up LSM as part of the core interventions for mosquito control activities within their NMCPs. Both countries are raising local funding from their governments to implement LSM operations including larval source reduction, environmental modifications, and larviciding operations. Tanzania has developed local manufacturing capacity for biorational larvicides to address supply chain gaps associated with international procurement [51]. As a result, the two countries offer great promise as places where piloting of the mosquito abatement model could be rolled out and intensely evaluated for evidence on operational feasibility in the greater African continent.

### Differences between the US mosquito abatement programmes and mosquito control programmes in Africa

Larval habitats in the US range from large water bodies such as swamps and marshlands, woodland pools, temporary pools, ponds created by melting snow, floodplains along streams and riverbanks, irrigated fields, rice paddies, and meadows, to smaller habitats such as containers, tree holes, tires, cemeteries, and holes in the rocks. Much of this is similar to aquatic habitats in SSA where the main vectors of malaria colonize ephemeral, freshwater habitats, including floodplains and irrigated areas (*An. gambiae *sensu stricto*, Anopheles coluzzii* and *Anopheles arabiensis*) [52–54] in addition to more permanent aquatic environments (*An. funestus*) [55, 56]. Some places in the US also contented with brood mosquitoes near populations centres [57], where millions of adults emerge synchronously requiring adulticide interventions.

Some of the observed major differences between mosquito control programmes in SSA and the US are:

1. Mosquito control efforts in the US are primarily targeted on outdoor control, as widespread house screening and air conditioning has excluded mosquitoes from indoor environments and eliminated the need for indoor mosquito control. In SSA, however, adoption of improved housing characterized by window, door, and eave screening as a major public health intervention against mosquitoes has been slow [58–60], with a few exceptions such as in the case of the city of Dar es Salam, Tanzania, where it is reported that between 2004 and 2008, there was a rapid adoption of house screening primarily motivated by nuisance biting and convergence of market forces facilitating growth of horizontal distribution channels for screens [60]. This coupled with highly competent anthropophilic mosquito vectors that rest indoors, has meant that the two main widely implemented vector control tools in the continent, IRS and LLINs, are primarily designed for indoor control [61], where mosquitoes and humans are likely to interface [62, 63].
2. In the US, control of nuisance mosquitoes is a substantial operational goal in addition to disease control, whereas in Africa, government-implemented mosquito control is primarily for disease control.
3. In the US, mosquito control benefits from decentralized management and funding leading to highly stratified and targeted implementation allowing room for more innovation. Indeed, in the US many larger abatement districts may have full time researcher(s) on staff allowing them to generate local evidence, develop and assess local innovations and solutions. In addition to a great spatiotemporal, and ecological heterogenicity that informs local decision-making, the US has a great diversity of economic resources depending on locality, as well as a political and cultural diversity which is expressed in local acceptance to mosquito control on private property and use of pesticides, as examples.

In SSA, mosquito control is largely donor funded and centrally implemented following WHO guidelines and recommendations that are applied uniformly across the board, oftentimes not taking into account contextual heterogeneities of countries. In contrast, there is more flexibility on how mosquito control operations are driven in the US, the tools available for control are also diverse, allowing for contextualization and community driven control approaches.

(4) Mosquito control programmes in the US emphasize integrated vector management, which often include sizeable component of LSM, and as such, many programmes have larval control components constituting equal or sometimes greater proportion of the entire mosquito control operations when compared to adulticiding. The US control programmes target diverse larval habitats, including micro habitats such as vehicle tires, and apply sophisticated GIS tools to map the habitats, thus, leading to rapid generation of precision data on larval habitats to target for LSM operations. Conversely, in most vector-borne disease endemic SSA countries context, larvae control is implemented at limited scale, in a few countries, for a myriad of reasons already cited. Subsequently, the features of the integrated approach observed in the abatement districts visited are discussed here, and the lessons for vector-borne disease endemic SSA countries suggested.

### Integrated vector management: lessons for sub-Saharan African countries’ mosquito control programmes

#### Community outreach and education

Education and outreach activities need to form part of the integrated mosquito control strategy in Africa. Generally, the communication strategies and media outlets used in the US, such as interviews in newspapers, scientific publications, or quarterly, or annual reports will not reach a large part of the target population in Africa due to the high level of illiteracy, particularly in rural communities as well as the poor access to these communication media. It is imperative, therefore, that communication strategies are adapted to the local context to ensure maximum coverage. These may include meetings with community members, outreach to specific community groups such as the youth, women, religious leaders, politicians, and other organized groups. The use of social media is rapidly increasing even in rural parts of SSA and presents a unique opportunity for additional outreach. Importantly, school-based communication strategies have been shown to result in positive behaviour change in the students’ family and should be encouraged [64]. Many US abatement programmes have also developed well-received outreach programmes for children which should be emulated [65].

#### Diseases surveillance

In the US, vector borne disease surveillance is done to estimate the risk of transmission of diseases such as West Nile encephalitis, EEE, SLE, and dog heartworm [66, 67]. Pathogen surveillance in mosquitoes is often more centralized and the testing is done proactively (often weekly) rather than reactively, thus, a monitoring tool enabling early warning and prompt response before human cases appear. Mosquito collections are done using diverse trapping methods, including gravid traps, CDC or New Jersey light traps (NJLTs), and Biogents BG-Sentinel traps, targeting various mosquito species and behaviours. The bulk of vector control surveillance in SSA is aimed at malaria disease. Additional vector and pathogen surveillance is often conducted reactively in response to disease outbreaks, even in places where outbreaks commonly occur [50]. There is a need to set up or increase the number of sentinel, as well as spot check surveillance sites for vector borne diseases in areas at risk of disease to inform control in advance of disease outbreaks in a concerted one-health approach using community-based approaches [68] for cost savings and efficiency.

#### Larval source management

##### Larval source reduction

Source reduction offers a long-term, sustainable way to target mosquito populations in their aquatic habitats. Larval source reduction consists of physical and permanent removal of mosquito aquatic habitats through a number of actions including: dumping water from containers or installing catch basin drains to prevent the creation of standing water collections; reaching out to community residents to rid their home environment of unwanted artificial containers or trash; installing screens to water harvesting containers; maintaining roof gutters flowing to avoid them becoming aquatic habitats; changing bowls and bird baths twice weekly among other methods [1]. It also includes collecting used tires which are then recycled, preventing them from ending up abandoned in the environment. In the US, some abatement programmes work closely with the departments of public works to assist with drainage of stagnant water which is a practice that obviously could work in the SSA context (historically, some abatement districts were even issued dynamite for the purposes of draining water !) [1]. In the SSA scenario, local public health departments could be sensitized on the potential negative impact of creating larval habitats during road construction, farming and other land use activities, and could assist in drainage of large standing water and unclogging of drainages to prevent mosquito breeding. In some US jurisdictions, mosquito control authorities have legal authority to mandate, or directly administer larval source management on private property.

##### Larval control

Larval control is a significant strategy used by US mosquito abatement programmes [16, 69]. It consists of the regular treatment of water sources with larvicides. Targeting larval stages is a very effective strategy in the sense that the larval stages of mosquitoes are usually constrained within specific habitats, are more accessible compared to the adult stages, and are more vulnerable to different larvicidal operations targeted at them. Different larvicides are used by the programmes that include, biorational larvicides made from *Bacillus thuringiensis* and/or *Bacillus sphaericus* and *Saccharopolyspora spinosa* formulations*,* insect growth regulator (IGRs), and oil/surfactants. Different formulations including granule, liquid and briquettes are used depending on the type (including ephemerality) of breeding habitats. These larvicides are options available for SSA, and depending on the larval ecology and vector species targeted, these options should be explored to supplement mosquito control efforts especially for exophilic, zoophilic and day biting mosquitoes [1].

##### Environmental management

From a historical standpoint, environmental manipulation and modification to make the environment less conducive to mosquito larval productivity has been a significant component of mosquito control in the US [1, 17]. Environmental management requires full understanding of the mosquito ecology, population dynamics and behaviour to be effective [70, 71]. Draining of ditches, swamps and marshes to ensure that water is lotic, or completely drained out, or impounding marshland waters, clearing storm drain sewers and catch basins of organic debris, clearing the edges of detention ponds and other stagnant water bodies, building of dykes and levees, correction and straightening of waterways, are among key measures that implemented in collaboration with public works agencies in the US to ensure that the environment is generally less conducive to mosquito larval productivity. Other environmental management approaches include implementing agricultural management practices that make the agricultural lands less conducive to mosquito productivity, such as ensuring that irrigation channels are lotic, and do not have vegetation overgrowths. Setting of residential buildings away from marshlands and screening of houses also constitute environmental management practices that have been historically implemented by the US mosquito control agencies [70, 71].

#### Adult mosquito control

Because adult mosquitoes are mainly restricted to outdoor environments in the US, adult mosquito control activities are based on the use of fogging techniques including the use of both gas-powered and electric ultra-low volume (ULV) machines that allow insecticide droplets to be dispersed from the spray unit to the environment [72]. This measure is in some places reserved as an emergency method when adult densities become very important and cannot be controlled by other measures. ULV applications are done with small particle insecticides and after sunset when honeybees and other pollinators are not foraging to minimize the impact of these measures on non-target species [73]. As SSA countries target malaria elimination, such methods could be applicable to arrest disease outbreaks and re-introductions, as well as urban settings (in response to *An. stephensi*, but must be implemented cautiously and as a last resort to minimize impact to the environment). Importantly, safe outdoor control of mosquitoes still requires more innovation and needs to be the focus of ongoing research.

#### Adoption of advanced technologies

Multiple new innovations can tremendously improve the quality and effectiveness of mosquito control programmes. These innovations can be used to improve the management of field operations, such as use of GIS; digitalized data collection, such as the use of tablets; data visualization and reporting, through dashboards and automated reports and control; and using drones for mapping as well as application of larvicides. These will improve resource tracking and accountability and ensure the cost effectiveness of the mosquito programmes [74]. The teams visiting the US abatement districts witnessed particularly exceptional examples of these technologies working together where smart (web connected) traps record an increase in mosquito abundance above an actionable level, therefore, a control operation is planned in the area, technicians receive their orders on their tablets and release the drone which uses precision pre-determined GIS guided deployment of pesticides to only areas of known standing water in the flight path. A database automatically receives reports from the drone of the exact quantity and location of pesticides applied. While this example may seem unrealistic for many SSA mosquito control programmes, parts of this might immediately be relevant for adoption within specific local contexts, especially those implementing larval control.

#### Capacity building

It is important to realize that the effective mosquito abatement programmes in the US are run on the backdrop of a huge investment in technical as well as infrastructural capacity. Most of these programmes have functional and serviced trucks and equipment for the deployment of the insecticides and surveillance (Fig. 2, Table 2). Some of these programmes have planes and drones of various sizes for the application of insecticides and larvicides as well as for surveillance (e.g., of standing water). These programmes are supported by intentionally designed surveillance and monitoring platforms which receives current data through frequent sampling.

Mosquito control programmes in SSA will need the initial investment to set up equally capable platforms that address mosquito control needs at the sub-national level, but importantly, will need to identify sustainable avenues for funding their recurrent expenditure to ensure that such huge investments do not end up being redundant. It is important to note that many of the US abatement programmes started as community initiatives because of the need for mosquito control and continue to have community representation in their boards of management. Importantly, there will be a need to show the value of investing in building and sustaining such capacities and, therefore, it is crucial that data is collected, analysed and disseminated at all levels of government and local communities, and scientific metrics applied to estimate, the burden of disease averted where necessary. For sustainability, it will be important to understand what is implementable using locally available community systems such as community health volunteers (CHVs) and the level of supervision required to ensure maximum impact. Well trained technical staff will be required at the sub-national district level to drive and sustain community-based entomological surveillance and control operations, and, therefore, the programmes have to be designed in a manner that attracts and retains such cadre of staff, including providing a clear pathway for career growth.

## Conclusions and call to action

There are important takeaways the observations of the US mosquito control programmes which could contribute directly to SSA malaria control programmes. The decentralization of mosquito control programmes at the sub-national level, to the districts, counties and villages, would ensure the programmes are tailored to local contexts in which they operate and explore local funding, innovation, and sustainable models. Some recommendations based on these experiences are:

(i) There is need for SSA countries to re-centre their IVM programmes, and here the authors suggest that more countries should conduct operational research studies on LSM to generate additional evidence that will inform operationally impactful contexts in which LSM could be applied in vector-borne disease endemic SSA countries. Such data needs to be generated in several diverse ecological and land use settings to ensure the level of impact is assessed properly.
(ii) Community centred integrated vector management (IVM) and surveillance approaches need to be considered as there is potential for high levels of acceptance, engagement and sustainability. Members of the community are likely to be more dedicated to the IVM campaigns as means to earn income and improve the economic well-being of the communities. Community involvement will also benefit from local knowledge for larval habitat surveillance, mapping, removal, larviciding applications and adult control, thus make mosquito control more sustainable and enjoy community buy-in and support.
(iii) Higher adoptions of LSM within the context of integrated approach could offer opportunities for the innovative manufacture and distribution of larvicides within the continent. This has several multiplier effects including creating sustainable larviciding value chain, employment opportunities for local communities in the disease endemic areas, hence enhanced adoption.
(iv) The place of house improvement needs to be re-evaluated and re-emphasized. The two major interventions, LLINs and IRS, focus on indoor control of adult mosquitoes that rest and feed indoors. African mosquito control professionals need to partner with their professional counterparts in the public works and the built environment, and country legislatures to enact laws that will progressively promote house screening as mandatory part of building code. This will eliminate the need for indoor control and, therefore, redirect the meagre resources to outdoor control. It will also reduce residual transmission that occurs indoors while human beings are not sleeping under bed nets and hence exposed to infectious bites.
(v) African governments need to re-evaluate the funding model for vector control and shift away from the heavy donor dependence to locally mobilized funding. This has the advantage of allowing countries to tailor interventions to local contexts and not implementation of solutions recommended by the funding partners, or that align with funding partners’ interests. Sub-Saharan African countries can also explore innovative funding models, based on local taxation opportunities that explore taxable items or ventures that could generate resources for mosquito control at the local level without increasing overall tax burden. These could include including mosquito control budget line in the local municipal business permits, among other options, and ring-fencing the funds exclusively to mosquito control.
(vi) The current scenario is such that public entomology is secondary to disease epidemiology and, therefore, most of the resources are allocated to case management to the detriment of vector control efforts even as empirical evidence indicates that vector control has contributed to more than half of the reduction in malaria burden [75]. While case management and epidemiology are vital, public health entomology and vector control efforts could contribute significantly to burden reductions as was observed between 2000 and 2015 [5]. Meticulously devolved vector control approaches in addition to case management could see significant declines in disease burden.
(vii) The centralized model of mosquito control, where all decisions about funding and intervention deployment is made at the centralized MOH level and implemented at the community level without input by the local communities, needs to be re-evaluated. The US abatement model is devolved to cover defined finite geographical areas where they are able to conduct adequate surveillance and control. Countries in SSA could also develop their vector control operations, increase their staffing and equip them adequately to respond to vector borne disease at the local context. This would create work opportunities for members of the local community while leveraging local knowledge for vector control.
(viii) Disease endemic countries in SSA must encourage stronger cross-border collaborations on vector surveillance and control. The contexts of vector control in many SSA countries are similar, with similar vectors, similarities in their ecologies, structural organization and funding situation. There are many dividends to be realized from stronger pan-African collaboration in vector control, rather than working in silos. This will provide opportunities to create synergies in sharing data and information on vector control, optimizing resource use, sharing expertise, especially with countries where entomological capacity is still limited, and ensuring that the vector map continues to shrink through concerted control efforts.

## Data Availability

Not applicable.
